# Asymmetric dimethylarginine compartmental behavior during high-flux hemodialysis

**DOI:** 10.1080/0886022X.2020.1797790

**Published:** 2020-07-30

**Authors:** Qiuna Du, Jiayuan Gao, Renhua Lu, Yun Jin, Yanfang Zou, Chen Yu, Yucheng Yan

**Affiliations:** a Department of Nephrology, Tongji Hospital, Tongji University School of Medicine, Shanghai, China; b Department of Nephrology, Renji Hospital, Shanghai Jiaotong University School of Medicine, Shanghai, China

**Keywords:** Hemodialysis, hemodynamics, uremic toxins, compartmental behavior

## Abstract

**Aim:**

The accumulation of uremic toxins, such as asymmetric dimethylarginine (ADMA), has emerged as one of the major cardiovascular disease-related risk factors in patients with end-stage renal disease (ESRD). Based on the low molecular weight of ADMA, hemodialysis (HD) should theoretically effectively remove ADMA. In this study, we investigated the clearance behavior of ADMA during high-flux HD.

**Methods:**

Eight HD patients without residual renal function were included. Blood samples were collected at 0, 30, 60, 120 and 240 min after dialysis started, as well as 1 h and 48 h after dialysis. ADMA level was detected by HPLC-MS/MS. Herein, the ADMA level in blood cells and the ADMA protein binding rate were measured. Accordingly, the dialyzer extraction ratio was also determined.

**Results:**

The reduction ratio (RR) of ADMA (corrected for hemoconcentration) was significantly lower, at only 37.21 ± 6.44%, than that of urea and creatinine (*p* < .05). Interestingly, its clearance from plasma was precipitous early in dialysis and became slowly from 60 to 240 min. Additionally, a greater inlet erythrocyte than plasma concentration was found for ADMA. The dialyzer extraction ratio was comparable between ADMA and creatinine or urea (83 ± 5% for ADMA vs. 84 ± 3% and 88 ± 2% for creatinine and urea, respectively; both *p*>.05). Urea and creatinine had a slight rebound ratio of less than 10% at 1 h after the completion of HD. In contrast, considerable rebound of approximately 30% was detected in ADMA.

**Conclusion:**

This study suggests that ADMA may present a multicompartmental distribution that cannot be representatively reflected by the urea kinetics model.

## Introduction

Cardiovascular disease (CVD) is the leading cause of mortality in end-stage renal disease (ESRD) patients with an incidence of 10–20 times higher than that in the general population [1]. Traditional risk factors, including diabetes mellitus, hypertension, obesity, dyslipidemia, and smoking, do not completely account for the excess CVD in this patient group. Indeed, other factors, including the accumulation of uremic toxins, such as asymmetric dimethylarginine (ADMA), have emerged as major risk factors in patients with ESRD [2–4].

Nitric oxide (NO) is derived from the metabolism of L-arginine [5]. It is considered to play a protective role in the cardiovascular system because it inhibits vascular muscle cell proliferation and the adhesion of monocytes to the endothelium [2]. The enzyme NO synthase (NOS) can be inhibited by endogenous methylarginines, and ADMA is considered to be one of these endogenous NOS inhibitors [5]. Substantial evidence has shown that increased plasma concentration of ADMA is consistently related to cardiovascular complications in ESRD patients [2,6–8].

ADMA is a guanidino compounds with a low molecular weight of 202 Da, Theoretically, hemodialysis (HD) should be very effective in eliminating this endogenous NOS inhibitor in the blood. However, this may not be the case, as pointed out by many studies reporting an ADMA reduction ratio between 20 and 40% during maintenance HD [9–12]. Eloot et al. [13,14] also demonstrated that the kinetic behavior of some small water-soluble guanidino molecules is different from that of urea during HD. In addition, it has beesuggested that [15,16] red blood cell (RBCs), which may act as modulators of the plasma ADMA level, cause the presence of substantial quantities of protein-incorporated ADMA.

To the best of our knowledge, the intradialytic kinetics of ADMA has not been thoroughly studied during high-flux HD. To gain better insights into the kinetics of ADMA as an intracellularly sequestered solute, we investigated the level and clearance behavior of ADMA in stable maintenance HD patients. Erythrocytes are virtually the only easily accessible intracellular compartment, and the compartmental distribution between plasma and erythrocytes was examined in this study. We elucidated that the clearance behavior of ADMA may differ from that of urea.

## Materials and methods

### Patients and dialysis prescription

A total of 8 stable maintenance HD patients without residual renal function were included in this study. Patients less than 18 years old, with diabetes mellitus, systemic lupus erythematosus or hemolytic disease, with less than 3 months on HD, with hospitalization or an acute illness within 1 month, with changes in the dialysis prescription within 1 month before sample collection, and treated with erythropoietin or estrogen within the last 3 weeks were excluded. All patients had been regularly dialyzed three times a week with high-flux dialyzers (FX60, Fresenius) for 4 h per session. A delivered spKt/V dose reached at least 1.4 in the last 3 months, as calculated by the Daurgirdas’ second-generation equation [17]. A constant dialysate flow rate of 500 mL/min and blood flow rate of 250 mL/min were applied for a total treatment of 4 h. All subjects were provided with a standard low-protein diet before the onset of the investigative dialysis treatment. During the investigative dialysis session and in the subsequent one hour, patients remained in bed, and no food or beverage were allowed. The investigative dialysis treatment was defined as the first HD session after the longest dialysis interval of a week [18,19]. This study was undertaken in accordance with the Helsinki Declaration and was approved by the hospital ethical committee, and written informed consent was obtained from all subjects and the clinical trial was registered at http://www.chictr.org.cn/index.aspx (ChiCTR1800015368).

### Sample collection and analysis

The predialysis blood sample was obtained from arteriovenous fistula before the infusion of saline or heparin and taken from the inlet and outlet blood lines 30, 60, 120 and 240 min after starting dialysis while stopping ultrafiltration and maintaining the blood pump. Venous blood was taken from the arm of contralateral arteriovenous fistula 1 h after the end of dialysis. It was assumed that 1 h was sufficient for the equilibration of fluid between the plasma and the remainder of the extracellular compartment. An additional blood sample was obtained immediately prior to the next HD session (∼48 h later) in patients. Two-milliliter blood samples were immediately centrifuged at 3000 rpm for 10 min, after which the plasma was stored at −80 °C until analysis. And additional 1-mL blood sample was then subjected to lysis by three freeze-thaw cycles with 1 min of freezing in liquid nitrogen and 5 min of thawing in a 37 °C water bath. The blood lysis appeared complete as judged by the minimal to absent sediment using hematoxylin and eosin staining.

The concentrations of urea, creatinine, albumin, hemoglobulin were determined by an automatic biochemistry analyzer. Hematocrit was measured using freshly drawn blood at various time points during and after HD. To correct plasma solutes level due to hemoconcentration by ultrafiltration, the ratio of serum albumin after versus before dialysis was calculated [20].

Sample ADMA level was measured by high-performance liquid chromatography coupled with tandem mass spectrometry (HPLC-MS/MS) [21,22]. For whole blood ADMA level determination, the whole-blood lysate was thawed, 50 µL of the sample was transferred to an Eppendorf tube, and 50 µL of 10% trichloroacetic acid was added for deproteinization. The sample was left on ice for 10 min and then centrifuged. The supernatant was used for determination of the whole-blood ADMA concentration. The ADMA concentration in blood cell (basically erythrocytes) was determined after subtraction of the plasma contents from the whole-blood contents, taking into account the volume occupied by packed cells, which was based on instantaneous hematocrit determinations, as follows [19].

ADMA concentration in RBCs = [ADMA concentration in whole blood lysate – ADMA concentration in plasma × (1 – hematocrit)]/hematocrit

To detect the binding state of ADMA to plasma protein, the protein binding rate was measured (*n* = 23). Briefly, the plasma were introduced into the ultra-centrifugal filter of a 10-kD molecular weight cutoff membrane (Pall, USA) and then centrifuged [23]. And the protein binding was also determined by means of equilibrium dialysis against phosphate buffer PH = 7.4 [24].

### Calculation of dialyzer extraction ratio

To determine the removal capacity of the solutes through dialyzer, the clearance by dialysis irrespective of the blood flow rate was assessed. From the inlet and outlet plasma concentrations, C_plasma inlet_ (C_pi_) and C_plasma outlet_ (C_po_), the dialyzer extraction ratio (E) was calculated considering diffusion [14]:

$$E=\left(C_{\text{pi}}-{\;C}_{\text{po}}\right)/C_{\text{pi}}\times 100\%$$

### Reduction ratio

Analogous to the definition for the urea reduction ratio, the ADMA reduction ratio (RR) can be defined as a function of the predialysis (C_pre_) and postdialysis (C_post_) concentrations [14]. C_post_ was corrected using the change in serum albumin.

$$\text{RR}=\;\left(1-{\;C}_{\text{post}}/C_{\text{pre}}\right)\;\times 100\%$$

### Effective solute removal

Due to redistribution in the body compartments following dialysis, the effective reduction ratio (RR_eff_) will be smaller than calculated with RR if a multi-compartmental distribution is present [13]. One hour was considered to be sufficient for reequilibration across the various compartments of the body. Additionally, measuring solute level 60 min postdialysis would most “truly” reflect the solute clearance. The concentration at 60 min postdialysis (C_60min post_) allowed calculating the RR_eff_ for the different solutes [13]. C_60min post_ was corrected using the change in serum albumin.

$${\text{RR}}_{\text{eff}}=\;\left(1-{\;C}_{60\text{min}\;\text{post}}/C_{\text{pre}}\right)\times 100\%$$

### Statistical analysis

Data were described as mean ± standard deviations. Repeated measurements were tested for statistical significance using ANOVA and the Scheffe’F test. Correlations between parameters were investigated by performing Pearson correlation analysis. Statistical significance was accepted for P less than 0.05. SPSS version 16.0 for windows software (SPSS Inc. Chicago, IL, USA.) was used for the statistical analysis.

## Results

### Patients’ demographics and hemodialysis characteristics

Eight male patients were included with the age of 50.71 ± 9.80 years old and average dialysis vintage of 128.86 ± 32.86 months. The causes of ESRD were that glomerulonephritis in 5 cases and unknown in the other 3 cases. The main characteristics of the patients and their dialysis sessions are shown in Table 1.

**Table 1.** Patient demographics and hemodialysis characteristics.

Parameters	Result
Age (years)	50.71 ± 9.80
Sex (M/F)	8:0
BMI (kg/m^2^)	22.21 ± 3.24
Hemoglobin (g/l)	141.42 ± 25.88
Hematocrit (%)	41.97 ± 8.46
Albumin (g/l)	41.81 ± 1.58
HD vintage (months)	128.86 ± 32.86
Body weight postdialysis (kg)	65.66 ± 10.13
Ultrafiltration Rate (ml/h)	753.50 ± 119.40
spKt/V	1.54 ± 0.18

BMI: body mass index; HD: hemodialysis.

### Plasma concentration of different compounds and erythrocyte level of ADMA

The concentrations of the studied compounds in plasma and erythrocyte were determined in various time points throughout dialysis (Table 2). In comparison to the predialysis inlet concentration, the plasma urea and creatinine levels continued to decrease over the course of dialysis, whereas there were no significant changes in the plasma inlet ADMA level from 60 min after starting dialysis (*p*>.05) (Table 2). Notably, the change in levels between the inlet and outlet was almost consistent for plasma urea, creatinine, and ADMA. A higher inlet erythrocyte than plasma concentration was found for ADMA. The erythrocyte inlet concentration did not alter significantly over time for ADMA, whereas there was a difference for urea (*p* < .05) (Table 2).

**Table 2.** Plasma and erythrocyte concentration at different times during and after dialysis.

	Plasma concentration (mg/l)			Erythrocyte concentration (mg/l)	
	BUN	Cr	ADMA	BUN	ADMA
0 min	804 ± 148	135 ± 24	0.243 ± 0.056	840 ± 149	0.73 ± 0.12
30 min Inlet	594 ± 121*	102 ± 20*	0.206 ± 0.053	642 ± 130*	0.69 ± 0.11
Outlet	65 ± 16^*†^	17 ± 4^*†^	0.044 ± 0.016^*†^	251 ± 65^*†^	0.70 ± 0.10
60 min Inlet	495 ± 108*	86 ± 18*	0.182 ± 0.047*	513 ± 141*	0.68 ± 0.10
Outlet	55 ± 17^*†^	14 ± 4^*†^	0.041 ± 0.014^*†^	236 ± 65^*†^	0.71 ± 0.11
120 min Inlet	387 ± 115*	68 ± 16*	0.173 ± 0.042*	402 ± 116*	0.66 ± 0.07
Outlet	44 ± 14^*†^	12 ± 5^*†^	0.029 ± 0.014^*†^	209 ± 58^*†^	0.64 ± 0.08*
240 min Inlet	228 ± 59*	49 ± 13*	0.163 ± 0.041*	335 ± 68*	0.65 ± 0.10
Outlet	28 ± 9^*†^	11 ± 6^*†^	0.037 ± 0.014^*†^	202 ± 41^*†^	0.64 ± 0.05*
Postdialysis 1 h	250 ± 67*	55 ± 13*	0.210 ± 0.049	366 ± 80*	0.67 ± 0.08
Postdialysis 48 h	729 ± 117	130 ± 16	0.219 ± 0.065	764 ± 171	0.72 ± 0.08

*p < 0.05 compared with predialysis concentrations; ^†^*p* < 0.05 compared with plasma inlet concentrations in respective time course. BUN: urea nitrogen; Cr: creatinine; ADMA: asymmetric dimethylarginine.

### Comparison of the ADMA and BUN solute clearance

Applying the solute concentration at 1 h postdialysis, the RR_eff_ values (corrected for hemoconcentration) of ADMA (17.60 ± 5.50%) were significantly lower than urea (71.33 ± 2.38%) and creatinine (67.41 ± 4.52%) (both *p* < .05). A compartmental distribution could be indicated by the finding that there was a significant difference between the RR and RR_eff_ values for urea and ADMA (both *p* < .05) (Table 3). Notably, ADMA was almost entirely free and not protein bound (89.79 ± 7.43%). The result was not different when using the equilibrium dialysis method to determine the protein-bound fraction.

**Table 3.** Reduction Ratio (RR) and effective reduction ratio (RR_eff_) for the different compounds (corrected for hemoconcentration).

Compound	MW (D)	RR (%)	RR_eff_ (%)
BUN	60	75.5 ± 1.99	71.33 ± 2.38^‡^
Cr	113	70.13 ± 4.38	67.41 ± 4.52*
ADMA	202	37.21 ± 6.44^*†^	17.60 ± 5.50^*†‡^

*p < .05 compared with BUN. ^†^*p* < .05 compared with Cr. ^‡^*p* < .05 compared with RR. BUN: urea nitrogen; Cr: creatinine; ADMA: asymmetric dimethylarginine;

MW: molecule weight; RR: reduction ratio; RR_eff_: effective reduction ratio.

**Figure 1.**
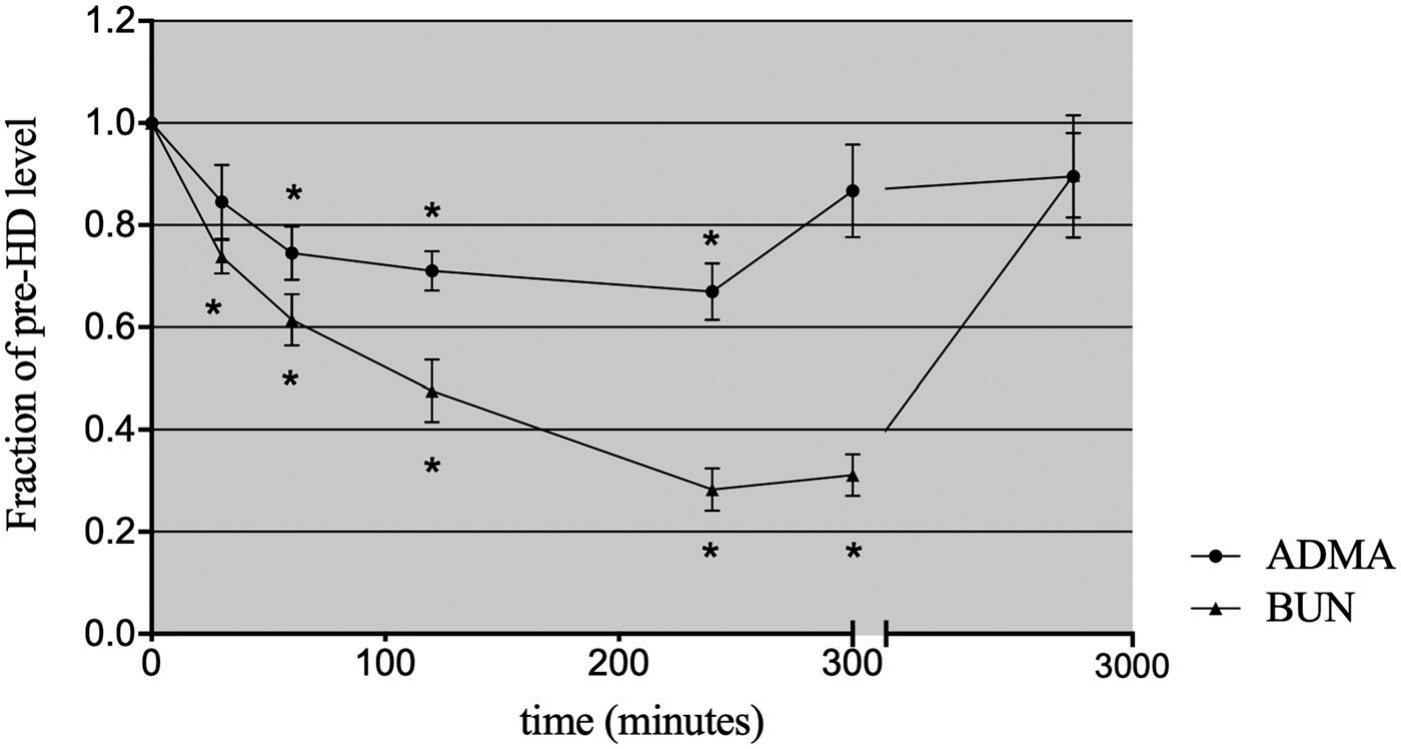
The mean solute levels of plasma ADMA and urea throughout the course of HD, 60 min postdialysis and prior to the next HD treatment (values are expressed as a fraction of the predialysis, initial plasma level). *p < .05 compared with the initial fraction of the predialysis level.

The tendency of solute clearance in plasma can be more clearly observed when depicted as a line chart (Figure 1). The urea and creatinine levels declined in an expected asymptotic manner during HD therapy. During the first hour of dialysis, the slope of ADMA in plasma was precipitous, similar to that for urea, and then became slower from 60 to 240 min (Figure 1).

**Figure 2.**
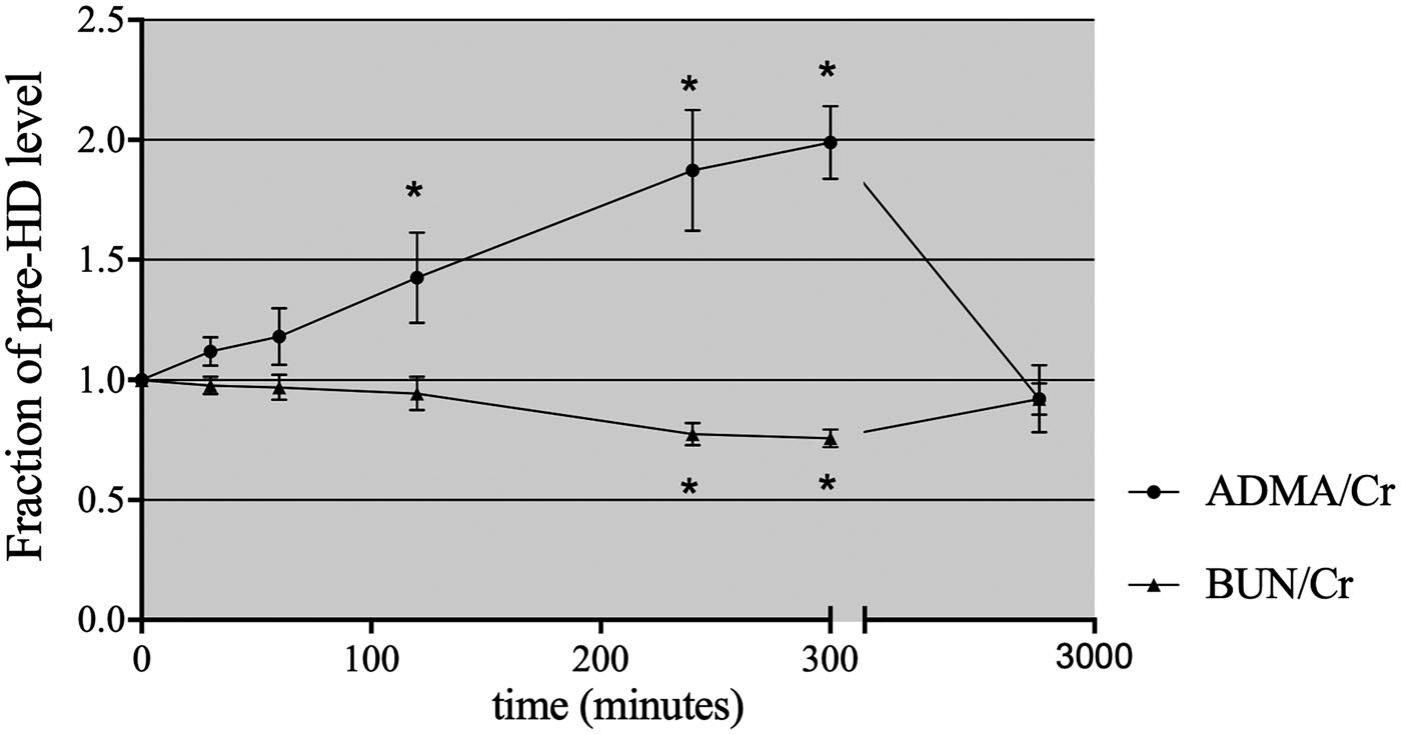
Time course of plasma ADMA/creatinine ratios during HD, 60 min postdialysis and prior to the next HD treatment. *p < .05 compared with the initial fraction of the predialysis level.

A slight rebound ratio less than 10% for urea and creatinine was detected at 1 h after completion of HD. The urea and creatinine levels remained low, at 31.59 ± 3.63% and 36.67 ± 4.83% of the predialysis values, respectively. In contrast, the ADMA level at 1 h postdialysis was significantly higher than that at the end of dialysis, at 86.68 ± 0.09% of the initial level, with considerable rebound of approximately 30%. At the beginning of the next dialysis session (48 h postdialysis), the urea and ADMA levels had returned to approximately 90% of the predialysis values (Figure 1). When the level of ADMA and urea was expressed relative to the serum creatinine concentration, the clearance behavior of ADMA and urea could be observed more clearly (Figure 2). The concentration of ADMA in erythrocytes remained constant after dialysis (Table 2).

Analysis showed that predialysis plasma ADMA level was positively correlated with hemoglobulin level (*r* = 0.779, *p* < .05) and erythrocyte ADMA level (*r* = .792, *p* < .05), whereas no correlation was found between predialysis plasma levels of ADMA and albumin (*p*>.05).

### Solute removal by the dialyzer

To assess the dialyzer solute removal efficiency during dialysis, the extraction ratio was calculated for different time points during dialysis. ADMA, creatinine and urea, defined as small soluble molecules, exhibited high dialyzer extraction ratio (the mean value of all time points: 83 ± 5% for ADMA vs. 84 ± 3% and 88 ± 2% for creatinine and urea, respectively; *p*>.05 for both ADMA vs. urea and ADMA vs. creatinine). This suggests that the dissimilarity in kinetic behavior among the different solutes cannot be explained by a difference in dialyzer extraction ratio during dialysis.

## Discussion

The present study aimed to evaluate the behavior of ADMA in stable HD and to compare it with a standard marker of dialysis adequacy, urea. Our study shows that the clearance of ADMA is significantly lower than that of urea and creatinine, and that the ADMA level rebounds to high plasma levels postdialysis. ADMA shows behaviors that are not well predicted by the behavior of urea despite being comparable in terms of a low molecular weight.

The dialyzer extraction ratio of ADMA did not differ from that of urea or creatinine and maintained a high value of approximately 83% during HD. As a consequence, the dissimilarity in clearance behavior between ADMA and urea and creatinine cannot be explained by inconsistent dialyzer extraction ratio. Kielstein et al. [25] found that removal of ADMA in standard dialysis was hampered because the compound is protein bound. In our study, we found that the ADMA protein binding rate was only approximately 10–19% using the multiple detection method, which is consistent with the findings reported by Tsikas et al. [26,27]. More likely, ADMA removal is hindered by complex kinetics and distribution.

In our study, dialytic removal resulted in an early and profound decrease in the plasma ADMA level at most during the first hour of dialysis. This indicated that only a very small amount of ADMA was present in the easily accessible plasma compartment. High differences in the ADMA concentration were detected between erythrocyte and plasma. This suggests that ADMA mainly existed intracellularly and was hardly removed, resulting in a smaller RR_eff_. Our data showed that plasma ADMA level immediately increased at 1 h after dialysis compared to that at the end of dialysis. The hemoconcentration did not play a role in this increase because there was no significant change in the total protein level during HD (data not shown). Others have noted similar plasma ADMA postdialytic rebound [28,29]. This large degree of rebound and the data presented suggests its kinetic behavior is different from that of urea. These data elicit the hypothesis that reequilibration of the plasma with other compartments, such as erythrocytes, is considered more likely to be responsible for the rebound effect, which contributes to the low effective removal of ADMA during HD.

For such sequestered toxin, the duration and frequency of dialysis may need to be increased to facilitate its whole-body elimination. Such seems to be the case with phosphate, another intracellular solute, slow nocturnal dialysis or short daily dialysis is highly effective in controlling serum phosphate [30–32]. Other study has found that peritoneal dialysis might be more effective on ADMA removal than HD and hemodiafiltration [33]. Finally, it should be noted that the presented data are based on the results of a single dialysis session for each investigated time schedule with a limited number of patients. Nevertheless, we found that ADMA behaved differently from solutes with comparable molecular weight, such as creatinine and urea, during HD.

In conclusion, the present study shows the removal behavior of ADMA during and after high-flux HD and suggests that not all changes in small soluble molecular concentrations in uremia and dialysis are representatively reflected by the kinetics of urea. Since increased ADMA blood concentrations are associated with cardiovascular complications in patients with ESRD, further understanding the dynamics of ADMA clearance in HD patients may help identify new removal strategies and improve the outcome of ESRD.
